# A Case of Herpetic Keratitis in an Orthokeratology Contact Lens Wearer

**DOI:** 10.7759/cureus.27388

**Published:** 2022-07-28

**Authors:** Hiroshi Toshida, Yoshinari Sadamatsu

**Affiliations:** 1 Ophthalmology, Juntendo University Shizuoka Hospital, Shizuoka, JPN; 2 Ophthalmology, Sadamatsu Eye Clinic, Saitama, JPN

**Keywords:** contact lens, orthokeratology, herpes simplex virus, herpetic keratitis, dendrites

## Abstract

We report a case of herpetic keratitis in an orthokeratology lens wearer. A 17-year-old man who wore an overnight orthokeratology lens for correction of myopia presented to our hospital with pain, lacrimation, and blurred vision affecting the left eye. His corrected visual acuity decreased to 18/20, and he showed dendrites and decreased corneal sensitivity in the left eye. The herpes simplex virus (HSV) immunochromatographic assay kit for the diagnosis of herpes epithelial keratitis was positive. As these findings were suggestive of HSV keratitis, topical acyclovir ointment was administered five times daily. All findings disappeared and visual acuity recovered to 20/20 at 14 days after the first visit. Herpetic keratitis rarely develops in orthokeratology lens wearers as well as contact lens (CL) wearers, although *Acanthamoeba* keratitis is sometimes erroneously diagnosed as herpetic keratitis in CL wearers with dendrites.

## Introduction

Orthokeratology involves the use of specially designed rigid gas-permeable (RGP) contact lenses (CLs) to correct mild myopia [1]. A highly oxygen-permeable material was utilized to enable continuous wearing during the night by individuals who preferred not to wear eyeglasses or CLs during the day. The principle of orthokeratology involves the alleviation of myopia by flattening the cornea with special lenses; however, some kinds of stress imposed on the cornea can lead to problems, such as corneal staining, microcysts, and microbial keratitis [1]. We report a rare case of herpetic keratitis in an orthokeratology lens wearer. To the best of our knowledge, this is the first reported case of such an association. All procedures performed in the study were in accordance with the ethical standards of the institutional committee and with the 2018 version of the Declaration of Helsinki [2] and its later amendments or comparable ethical standards.

## Case presentation

**Figure 1:**
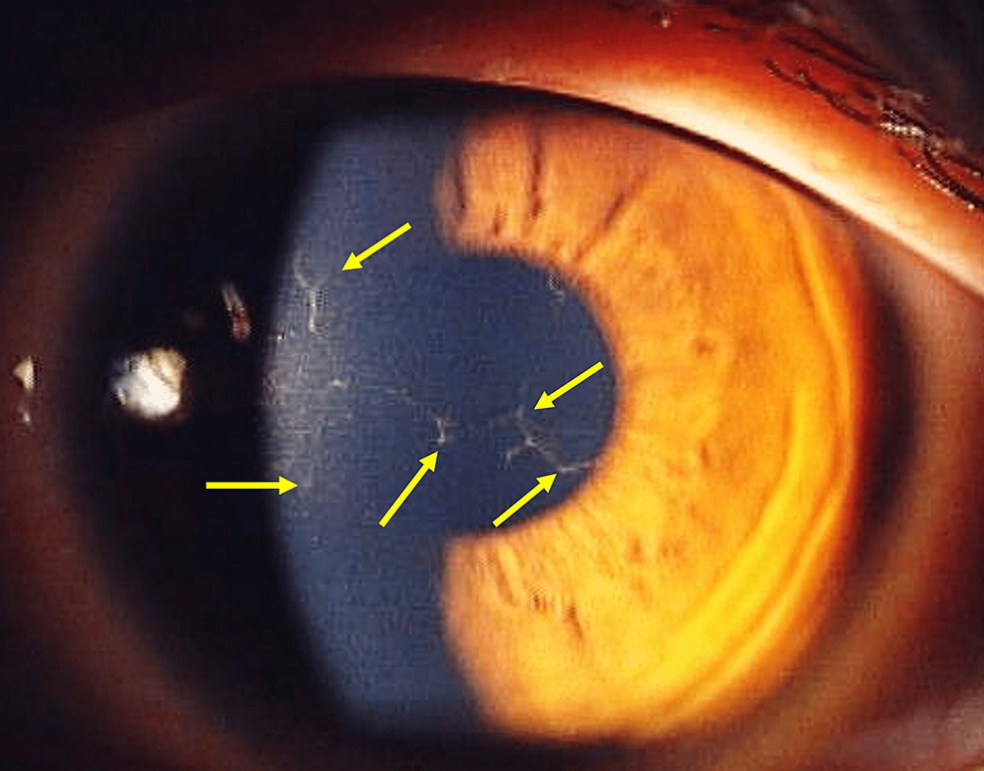
Slit-lamp image of the left eye at the first visit. Dendrites can be seen in the cornea (arrows).

A 17-year-old man presented with sudden eye pain, lacrimation, and blurred vision in the left eye. When referred to our clinic, the corrected visual acuity decreased to 18/20 in the left eye, while it showed no change (20/20) in the right eye. He had mild myopia and began wearing overnight orthokeratology lenses (My Emerald; Euclid Systems Corporation, Sterling, VA, USA) to correct his vision. The lenses were prescribed by another clinic three months ago. Because he was a high school student who belonged to a leading baseball team in the prefecture, he was unwilling to wear eyeglasses. Slit-lamp examination revealed dendrites (Figure 1).

**Figure 2:**
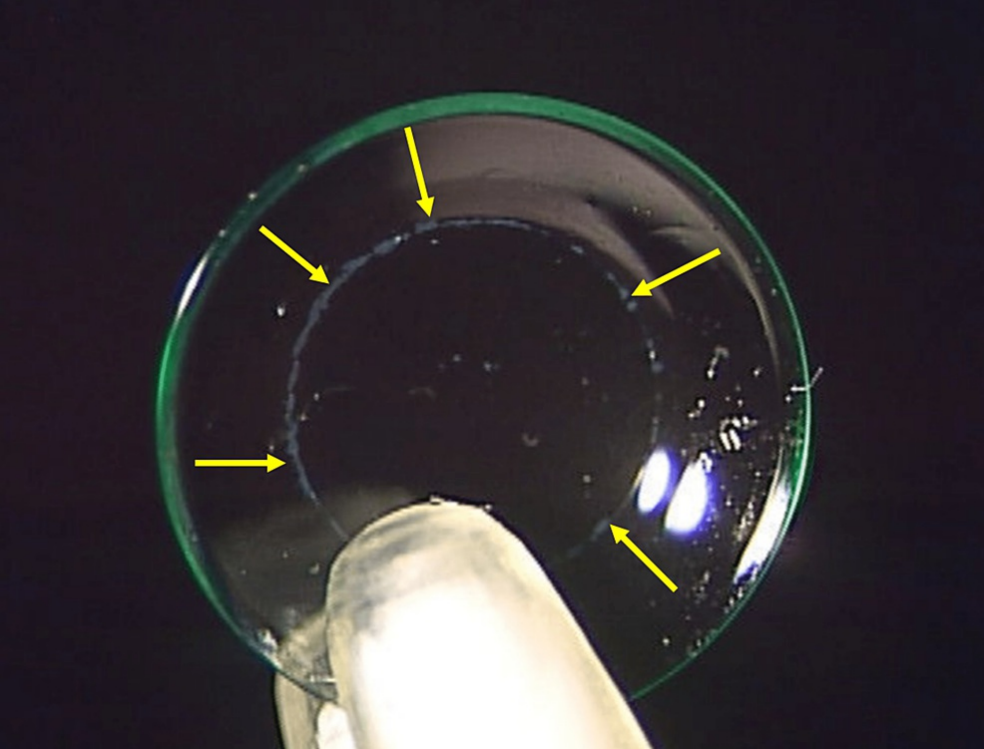
Deposits on the orthokeratology lens. Arrows highlight the deposits in the area of the reverse curve.

No conjunctival injection was observed in the left eye, and no findings were observed in the right eye. Corneal sensitivity measured with the Cochet-Bonnet esthesiometer in the center of the cornea [3] was decreased in the left eye (35 mm) than in the right eye (55 mm). His orthokeratology lenses were examined using microscopy, and deposits were observed on the lenses, especially in the area of the reverse curve (Figure 2).

**Figure 3:**
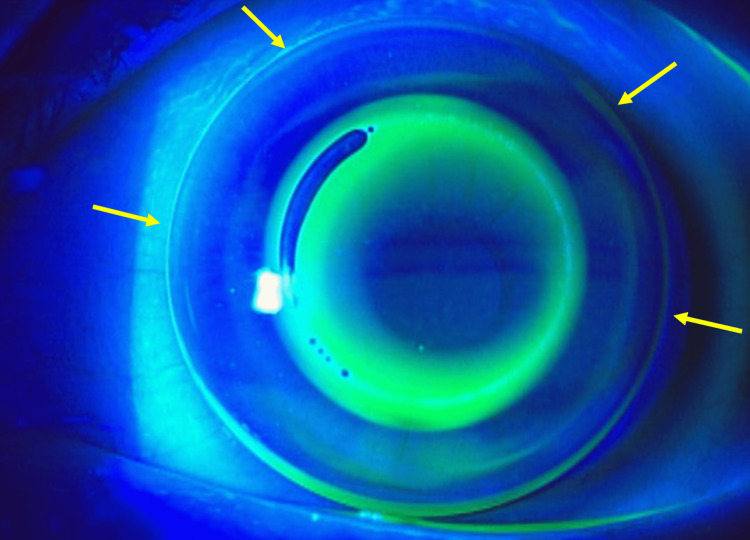
Lens fitting showing fluorescein pattern. It showed too steep lens fitting, especially the bevel was too narrow (arrows).

The fitting pattern in the left eye was extremely steep, as shown in Figure 3.

**Figure 4:**
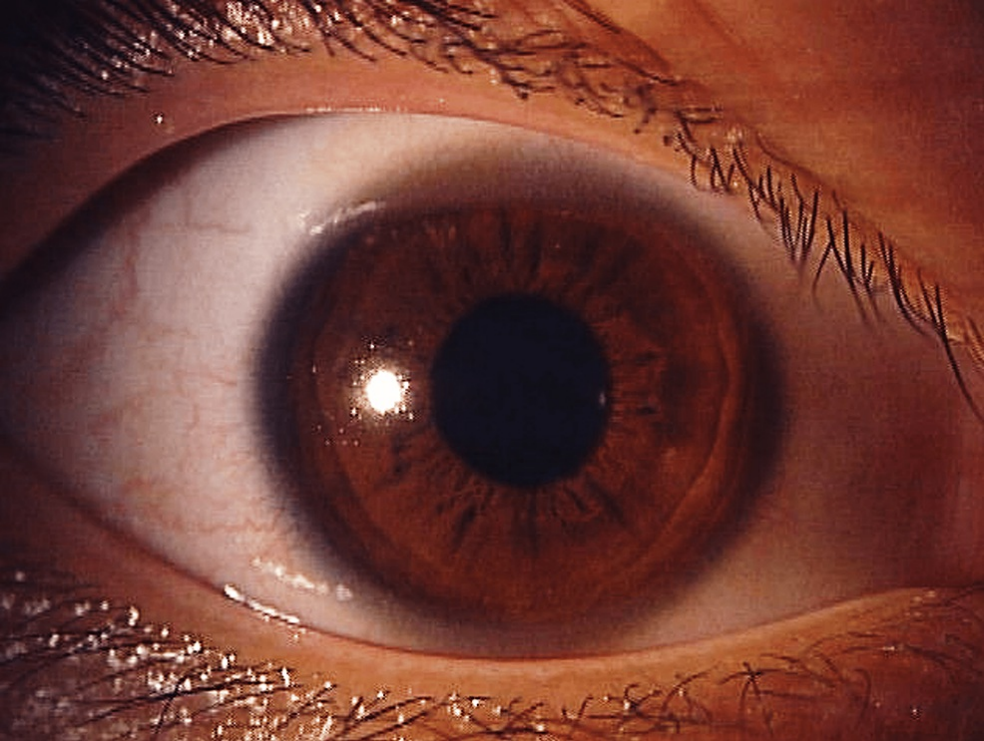
Ocular findings after treatment with acyclovir eye ointment. All findings disappeared in the left eye.

As these findings were suggestive of herpetic keratitis, topical 3% acyclovir ointment (Zovirax eye ointment, GlaxoSmithKline, UK) was administered five times daily. The herpes simplex virus (HSV) was then detected in corneal scraped samples using the HSV immunochromatographic assay kit for the diagnosis of herpetic epithelial keratitis (Checkmate Herpes Eye, Wakamoto Pharmaceutical Co., Tokyo, Japan) [4]. The medicine was effective, and the corneal findings disappeared (Figure 4). The visual acuity recovered to 20/20 in the left eye two weeks later. No recurrence was observed after treatment with topical acyclovir ointment.

## Discussion

Infectious keratitis is the most serious ocular complication associated with wearing orthokeratology lenses. *Pseudomonas aeruginosa* keratitis and* Acanthamoeba* keratitis are the major cause of serious CL-associated ocular complications not only in CL wearers [5] but in orthokeratology lens wearers as well [6-9]. *Acanthamoeba *keratitis can be erroneously diagnosed as herpetic keratitis [10,11]. Herpetic keratitis is caused by stresses such as trauma and is associated with wearing CLs [12]. It can also be caused by wearing orthokeratology lenses that reshape the cornea, thus placing a burden on the corneal epithelium. In the present case, checking corneal sensation and testing with an HSV immunochromatographic assay kit for the diagnosis of herpetic epithelial keratitis was helpful for proper diagnosis.

Unlike ordinary RGP lenses, overnight orthokeratology lenses are not worn to look through but are designed to improve uncorrected visual acuity by reshaping the cornea. Therefore, a higher priority is placed on therapeutic efficacy, and the prescription is less likely to be changed because the lens fit is poor. Eventually, the patient decided to discontinue wearing orthokeratology lenses because of herpetic keratitis and because the cost was too high, although the prescription could presumably have been changed to a different lens. Overnight orthokeratology lenses are designed to be worn during sleep when eye movements and blinking are decreased, so that the flow of the tear film under the lens decreases, resulting in hypoxia and the accumulation of waste products. Thinning of the corneal epithelium may occur when wearing orthokeratology lenses, creating a further burden at the cellular level [13]. Furthermore, because correctly cleaning orthokeratology lenses is more time-consuming than cleaning ordinary RGP lenses, the extent of lens cleaning often tends to be insufficient, and many wearers perform inadequate lens care. Cho et al. also reported that neither rubbing nor using either a one-step hydrogen peroxide solution or a povidone iodine-based solution is effective in removing stubborn deposits from orthokeratology lenses [14]. It is also important to visualize the lens deposits on the reverse curve as the area of the deposits [15]. It is possible that reshaping the cornea and dirty lens may have caused stress on the cornea and induced the onset of herpetic keratitis in the present case. Herpetic keratitis rarely develops in orthokeratology lens wearers and needs to be distinguished from *Acanthamoeba* keratitis, which is sometimes erroneously diagnosed as herpetic keratitis in CL wearers with dendrites.

## Conclusions

It has been suggested that wearing overnight orthokeratology lenses can induce the onset of herpetic keratitis. When we see patients with dendrites or pseudo-dendrites, it is important to diagnose herpetic keratitis, avoiding the erroneous diagnosis of *Acanthamoeba* keratitis in orthokeratology lens as well as CL wearers. In addition, checking corneal sensation and testing with the HSV immunochromatographic assay kit for the diagnosis of herpetic epithelial keratitis is useful for proper diagnosis.
